# Increased Serum Levels of Soluble TNF-α Receptor Is Associated With ICU Mortality in COVID-19 Patients

**DOI:** 10.3389/fimmu.2021.592727

**Published:** 2021-04-22

**Authors:** Esmaeil Mortaz, Payam Tabarsi, Hamidreza Jamaati, Neda Dalil Roofchayee, Neda K. Dezfuli, Seyed MohammadReza Hashemian, Afshin Moniri, Majid Marjani, Majid Malekmohammad, Davood Mansouri, Mohammad Varahram, Gert Folkerts, Ian M. Adcock

**Affiliations:** ^1^ Clinical Tuberculosis and Epidemiology Research Center, National Research Institute of Tuberculosis and Lung Diseases (NRITLD), Shahid Beheshti University of Medical Sciences, Tehran, Iran; ^2^ Department of Immunology, School of Medicine, Shahid Beheshti University of Medical Sciences, Tehran, Iran; ^3^ Chronic Respiratory Diseases Research Center, National Research Institute of Tuberculosis and Lung Diseases (NRITLD), Shahid Beheshti University of Medical Sciences, Tehran, Iran; ^4^ Department of Immunology, School of Medicine, Dezful University of Medical Sciences, Dezful, Iran; ^5^ Mycobacteriology Research Center, National Research Institute of Tuberculosis and Lung Diseases (NRITLD), Shahid Beheshti University of Medical Sciences, Tehran, Iran; ^6^ Division of Pharmacology, Utrecht Institute for Pharmaceutical Sciences, Faculty of Science, Utrecht University, Utrecht, Netherlands; ^7^ National Heart and Lung Institute, Imperial College London, London, United Kingdom; ^8^ Priority Research Centre for Asthma and Respiratory Disease, Hunter Medical Research Institute, University of Newcastle, Newcastle, NSW, Australia

**Keywords:** TNF-α, soluble TNF-α, COVID-19, cytokine storm, ARDS

## Abstract

**Background:**

Severe acute respiratory syndrome coronavirus 2 (SARS-CoV-2) that causes coronavirus disease 2019 (COVID-19) has infected over 112M patients and resulted in almost 2.5M deaths worldwide. The major clinical feature of severe COVID-19 patients requiring ventilation is acute respiratory distress syndrome (ARDS) possibly associated with a cytokine storm.

**Objectives:**

To elucidate serum levels of TNF-α and soluble TNF-Receptor 1 (sTNFR1) in patients with severe and mild COVID-19 disease as determinants of disease severity.

**Methods:**

We determined serum TNF-α and sTNFR1 concentrations in 46 patients with laboratory-confirmed COVID-19 (17 patients with severe disease within the intensive care unit [ICU] and 29 non-severe, non-ICU patients) and 15 healthy controls upon admission using ELISA. Subjects were recruited between March-May 2020 at the Masih Daneshvari Hospital Tehran, Iran.

**Results:**

Serum levels of sTNFRI were significantly higher in ICU patients (P<0.0001) and non-ICU patients (P=0.0342) compared with healthy subjects. Serum sTNFR1 were significantly higher in ICU patients than in non-ICU patients (P<0.0001). Serum TNF-α levels were greater in ICU and non-ICU patients than in the healthy subjects group (p<0.0001). The sTNFRI concentration in ICU (r=0.79, p=0.0002) and non-ICU (r=0.42, p=0.02) patients positively correlated with age although serum sTNFRI levels in ICU patients were significantly higher than in older healthy subjects. The sTNFRI concentration in ICU patients negatively correlated with ESR.

**Conclusions:**

The study demonstrates higher sTNFRI in ICU patients with severe COVID-19 disease and this be a biomarker of disease severity and mortality. Future studies should examine whether lower levels of systemic sTNFR1 at admission may indicate a better disease outcome.

## Introduction

Understanding the mechanism(s) and profiles of the cytokine storm observed in coronavirus disease 2019 (COVID-19) is crucial for the development of effective therapeutic interventions. Cytokine blockers and immune-host modulators are currently being tested in severely ill COVID-19 patients to cope with the overwhelming systemic inflammation (1). Elevated cytokine levels have been reported among severe COVID-19 patients indicating the therapeutic potential of existing immunosuppressive agents in COVID-19 (2).

COVID-19 is an infectious disease caused by severe acute respiratory syndrome coronavirus 2 (SARS-CoV-2) with symptoms such as fever, dry cough and shortness of breath (3). According to the disease state, patients are divided into two major groups; asymptomatic or mild cases that usually recover after showing mainly mild to moderate symptoms. However, severe cases (approximately 15%) develop multi-organ failure due to a cytokine storm resulting in respiratory failure. These cases require admission to an intensive care unit (ICU) and the disease can lead to respiratory failure and death (4, 5).

COVID-19 patients that require ICU admission have higher blood concentrations of IL-6, CXCL10, CCL2 and TNF-α as compared to patients with milder disease that does not require ICU admission (6). Most inflammatory mediators detected in patients with severe COVID-19 disease are probably produced by immune effector cells that are able to release large amounts of pro-inflammatory cytokines that contribute to the sustained systemic inflammation (7).

A pathogenic role for IL-6 and its receptor in COVID-19 has been described (8, 9). In contrast, a negative relationship between the concentration of soluble IL-2 receptor (sIL-2R) and T-cell number in the blood from COVID-19 patients has recently has been reported (10). TNF-α is produced primarily as a transmembrane precursor by monocytes/macrophages, but a number of other immune and structural cell types, such as T and B lymphocytes, mast cells, neutrophils, fibroblasts and airway epithelial cells also secrete this cytokine (11, 12). The TNF-α precursor is cleaved by TNFα-converting enzyme (TACE) to liberate TNF-α that acts by binding to two distinct membrane receptors on target cells: TNFR1 and TNFR2 (13).

TNFR1 is ubiquitously expressed within the lymphoid system and nearly all cells of the body, which likely accounts for TNF’s wide-ranging functions. TNFR2 expression is limited to certain lymphocyte populations including T-regulatory cells (T_regs_) (14). Generally, TNF-α binding to TNFR1 results in apoptosis due to the presence of death domains and binding to TNFR2 results in cell survival, although there is some degree of overlap depending upon the activation state of the cell and other factors (15).

Both TNFR1 and TNFR2 are released following cleavage of the membrane receptors by ADAM (a disintegrin and metalloproteinase) 17 (16) but the role of these soluble forms of TNFR1 and TNFR2 is debated as they bind to, and inhibit, TNF-α function during acute inflammation. In contrast, TNF-α/sTNFR1 complexes can act to enhance TNF-α function under chronic inflammatory conditions by the slow release of TNF-α (17). However, these effects may be concentration-dependent. In previous studies, circulating levels of sTNFR1 were associated with adverse outcomes and severity of myocardial infarction, mortality in subjects with a ruptured abdominal aortic aneurysm, the risk of death in patients with diabetic nephropathy and to adverse outcomes in diabetic patients (18).

We, therefore, compared the serum levels of sTNFR1 and TNF-α upon admission to Hospital in COVID-19 patients who either required ventilation in ICU or who had milder disease. We correlated the expression of sTNFR1 and TNF-α with clinical and biochemical markers including survival.

## Materials and Methods

### Patients

46 patients (17 ICU patients, 29 non-ICU patients; aged from 29 to 82 years) with confirmed COVID-19 and admitted into the General Hospital of Masih Daneshvari in Tehran from 5 March to 10 May 2020 were recruited. Patients came to the Hospital within 2-3 days of the onset of symptoms and were admitted immediately and samples obtained if positive for SARS-CoV2 by use of quantitative RT-PCR (qRT-PCR) of throat swab samples. Severe COVID-19 disease was confirmed by the presence of at least one of the following: respiratory rate≥30/min; blood oxygen saturation ≤93%; ratio of partial pressure of oxygen in arterial blood to the inspired oxygen fraction (PaO2FiO2) <300; lung infiltrates present on>50% of the lung field. Subjects admitted to ICU generally died 2-3 days following admission. Cytokine release syndrome (CRS) was determined according to the presence of clinical signs and symptoms (**Table 1**) and its severity was graded (19). All patients underwent a standard treatment protocol with antiviral medication and corticosteroids. Clinical and laboratory findings and chest X-rays or CT scans for all the patients were extracted from electronic medical records. In addition, 15 healthy subjects (aged 34 to 77 years), who came to the hospital for routine physical examination were also recruited.

**Table 1:** Demographic information and biochemical characteristics of severe (ICU) and mild (Non-ICU) COVID-19 patients.

	ICU (n=17)	Non-ICU (n=29)	Healthy (n=15)	^a^P value ICU & non-ICU	P value	
					non-ICU & Healthy	ICU & Healthy
Age, years (mean ± SEM)	66.2 ± 3.1 (39-81)	48.2 ± 2.8 (29-82)	54.73 ± 3.66 (34-77)	0.0002	0.1772	0.0227
Female n, % (mean age)	13, 76.5% (64.08 ± 3.839)	14, 48.3% (51.36 ± 3.489)	7, 46.66% (46.57 ± 3.854)	0.0213	0.4082	0.0089
Male (mean age)	4, 23.5% (73.00 ± 2.309)	15, 51.7% (46.00 ± 4.442)	8, 53.34% (61.88 ± 4.849)	0.007	0.0348	0.1531
Comorbidities (n, %)						
Hypertension (n=15)	10 (66.66%)	5 (33.34%)	–	0.004		
Chronic kidney disease (n=2)	1 (50%)	1 (50%)	–	0.696		
Diabetes (n=9)	5 (55.5%)	4 (45.5%)	–	0.197		
COPD (n=6)	2 (33.3%)	4 (66.7%)	–	0.844		
Cardiovascular disease (n=3)	1 (33.3%)	2 (66.7%)	–	0.893		
Malignancy (n=1)	1 (100%)	0	–	0.187		
High BMI (%)	7 (100%)	0	–	0.0002		
ARDS	1 (100%)	0	–	0.187		
Death; n, (%)	16 (94.11%)	0	–	<0.0001		
Erythrocyte sedimentation rate (ESR) (mm/hr) (95% Confidence intervals)	60.24 ± 7.92 (43.45-77.02)	50.72 ± 7.02 (36.34-65.11)		0.3921		
C-Reactive protein (CRP) (mg/l) (95% Confidence intervals)	27.00 ± 4.07 (18.38-35.62)	52.1 ± 21.4, n=28) (8.137-96.01)		0.3719		
Lactate Dehydrogenase (LDH) (U/L) (95% Confidence intervals)	759.2 ± 56.11 (640.3-878.2)	447 ± 32.5, n=28) (380.2-513.3)		<0.0001		
Troponin (pg/ml) (95% Confidence intervals)	0.020 ± 0.0006 (0.01934-0.02184)	0.0187 ± 0.0009, n=27) (0.01677-0.02057)		0.1343		
Creatine phosphokinase (CPK) (U/L) (95% Confidence intervals)	142.6 ± 17.83, n=16 (104.6 – 180.6)	113.1 ± 26.5, n=28) (58.80 – 167.5)		0.4381		

Values are presented as mean ± sem. 95% Confidence intervals are also provided in brackets.

a P values indicate differences between ICU and non-ICU patients. P <.05 was considered statistically significant.

ARDS, acute respiratory distress syndrome; COPD, chronic obstructive pulmonary disease; ICU, intensive care unit

The study protocol was approved by the Institutional Review Board for human studies of the Masih Daneshvari Hospital, Tehran, Iran and all patients provided written informed consent (IR.SBMU.NRITLD.REC.1399.123).

### Sample Collection and Cytokine Assay Analysis

Routine blood samples were obtained to examine blood count, coagulation profile, serum biochemical tests (including kidney and liver function tests), phosphocreatine kinase (CPK), lactate dehydrogenase (LDH), erythrocyte sedimentation rate (ESR), C-reactive protein (CRP) and troponin. Serum from whole blood was collected and the concentrations of TNF-α (R&D SYSTEM, Minneapolis, MN, US), IL-6 (R&D System) and TNFRI (ab100642, Abcam, Cambridge, UK) were measured by enzyme linked immunosorbent assay (ELISA) kit according to the Manufacturer’s protocols.

### Statistical Analysis

Analyses was performed using SPSS version 16.0 (SPSS, Inc. Chicago, USA) and GraphPad Prism software (version 6.07 Graph Pad Software, Inc.). P-values obtained from a parametric unpaired student t-test (mean ± SEM, 95% confidence intervals (CI)) was used for the variables with normal distribution. P-values <0.05 were considered as statistically significant. Non-parametric Mann-Whitney U test was used for non-normal distribution. Data in **Table 3** are analyzed using linear regression and R values are from Pearson’s correlation coefficient test.

## Results

### Demographic and Clinical Characteristics of Patients With COVID-19

Demographic data and clinical characteristics of the 46 patients with COVID-19 (ICU patients, n=17; non-ICU patients, n=29) and 15 healthy subjects are shown in **Table 1**. The mean age was 54.9 years (SEM; 2.46; range, 29 – 82 years) with those admitted to the ICU (both male and female) being significantly higher than in non-ICU group (66.2 ± 3.1 vs. 48.2 ± 2.8, p= 0.0002). Of the patients admitted to the ICU, 76.5% were female and 23.5% male. 16/17 (94%) of severe COVID-19 patients admitted to ICU died.

The levels of LDH in COVID-19 patients upon admission to ICU was higher than in less severe non-ICU COVID-19 patients on admission (759.2 ± 56.1 vs. 446.8 ± 32.5, P<0.0001) (**Table 1**). There were no significant differences in ESR, CRP, CPK and troponin between severe COVID-19 patients in ICU and milder patients not in ICU (**Table 1**).

### TNF-α and sTNF-RI Concentration Analysis

**Figure 1:**
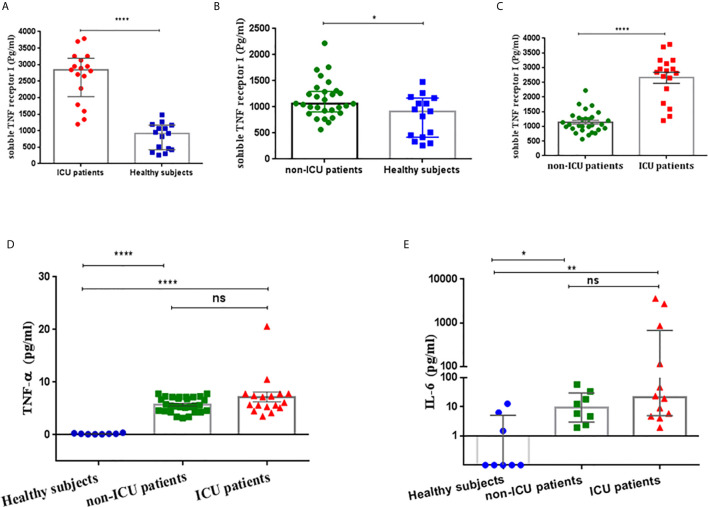
**(A)** Soluble TNF-α receptor I (sTNF-RI) concentrations in the serum of severe COVID-19 patients and healthy subjects. **(B)** Serum sTNF-RI concentrations in mild COVID-19 patients and healthy subjects. **(C)** sTNFRI concentrations in the serum of severe COVID-19 patients and mild COVID-19 patients. **(D)** Serum TNF-α concentrations in mild COVID-19 patients, severe COVID-19 patients and healthy subjects **(E)** Serum IL-6 concentrations in ICU patients, non-ICU patients and healthy subjects. *P<0.05, **p<0.01, ****P<0.0001 and ns, not significant.

Serum sTNFRI levels in severe COVID-19 patients on admission to ICU were significantly higher than in the healthy group (median; 2842 pg/ml, 5-95% percentile; 1197-3789 vs. 917.3 pg/ml, 5-95% percentile; 260-1479, p<0.0001, respectively) (**Table 2**, **Figure 1A**). There was a significant difference between serum sTNFR1 levels in patients with mild COVID-19 and healthy subjects (median 1061 pg/ml, 5-95% percentile; 633.4-1991 vs. 917.3 pg/ml, 5-95% percentile; 260-1479, p=0.0342, respectively) (**Table 2**, **Figure 1B**). Serum sTNFRI levels in severe COVID-19 patients on admission to ICU were significantly higher than in the mild COVID-19 patients (p<0.0001) (**Table 2**, **Figure 1C**).

**Table 2:** The concentration of sTNF-RI and TNF-α in COVID-19 patients and healthy subjects according to ICU status.

	ICU-patients n=17	Healthy n=15	P value	Non-ICU patients n=29	Healthy n=15	P value	ICU-patients n=17	Non-ICU patients n=29	P value
sTNF-RI (pg/ml)	2842(1197-3789)*	917.3 (260-1479)*	<0.0001	1061 (633.4-1991)*	917.3 (260-1479)*	0.0342	2654 ± 187^†^	1144 ± 66.58^†^	<0.0001
TNF-α (pg/ml)^†^	7.18 ± 0.94	0.200 ± 0.038 n=8	<0.0001	5.73 ± 0.26	0.200 ± 0.0380 n=8	<0.0001	7.18 ± 0.94	5.73 ± 0.26	0.0725
	ICU-patients n=12	Healthy n=8	P value	Non-ICU patients n=8	Healthy n=8	P value	ICU-patients n=12	Non-ICU patients n=8	P value
IL-6 (pg/ml)*	21.3 (1.94-3612)	0.1 (0.1-12.8)	0.0014	9.25 (1.94-58.9)	0.1 (0.1-12.8)	0.0135	21.3 (1.94-3612)	9.25 (1.94-58.9)	0.2454

^†^Values are presented as mean ± sem.

* Values are presented as median (5-95% percentile).

Serum TNF-α levels were similar between COVID-19 subjects upon ICU admission and non-ICU patients (7.18 ± 0.94 vs 5.73 ± 0.26 pg/ml, P=0.0725). However, both severe (7.18 ± 0.94 pg/ml, P<0.0001) and mild (5.73 ± 0.26 pg/ml, P<0.0001) COVID-19 patients had significantly higher serum TNF-α concentrations than healthy subjects (0.2 ± 0.03 pg/ml) (**Table 2**, **Figure 1D**).

Serum IL-6 levels were measured in a subset of the patients and healthy subjects were treated as a single group due to low numbers. IL-6 was significantly higher in both severe COVID-19 ICU patients [21.3 pg/ml, 5-95% percentile; 1.94-3612 pg/ml; p=0.0014)] and in non-ICU patients [9.25 pg/ml, 5-95% percentile; 1.94-58.9 pg/ml; p=0.0135)] compared with healthy subjects (**Table 2**, **Figure 1E**). However, there was no significant difference in IL-6 serum levels (p=0.2454) between severe and mild COVID-19 patients (**Table 2**, **Figure 1E**).

### Correlations Between TNF-α and sTNFRI With Clinical Variables in ICU/Non-ICU Patients

**Figure 2:**
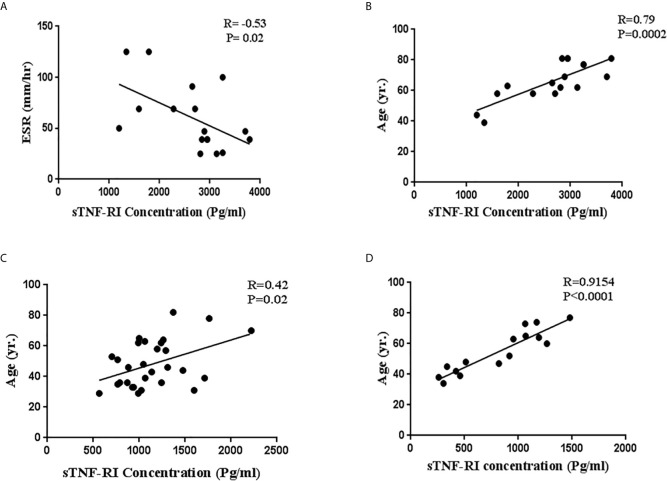
Correlations between serum soluble TNF-α receptor I (sTNFRI) levels in ICU patients with **(A)** ESR levels and **(B)** age and **(C)** between sTNFR1 levels and age in ICU patients and in non-ICU patients and between **(D)** sTNFRI levels and age in healthy subjects.

We next investigated the relationships between serum sTNF-RI and TNF-α level with LDH, ESR, CRP, troponin, CPK and age within ICU and non-ICU patients (**Table 3**). The level of sTNFRI in ICU patients was negatively correlated with ESR concentration (r=-0.53, p=0.02) (**Figure 2A**, **Table 3**). In contrast, the concentration of sTNF-RI in ICU (r=0.79, p=0.0002) and non-ICU (r=0.42, p=0.02) patients was positively correlated with age (**Table 3**, **Figures 2B, C**). There were no other significant correlations between serum TNF-α and sTNFR1 and any other parameters (**Table 3**). There was a significant correlation between serum sTNFRI and age within the healthy group (P<0.0001, r=0.9154) (**Table 3**, **Figure 2D**).

**Table 3:** Correlation between TNF-α and sTNFR and other factors in ICU and non-ICU COVID-19 patients and healthy subjects.

Patients	ESR		CRP		LDH		CPK		Troponin		Age		sTNFR1	
ICU	r	p	r	p	r	p	r	p	r	p	r	p	r	p
TNF-α	-0.42	0.09	-0.01	0.95	0.18	0.48	-0.41	0.10	-0.24	0.34	-0.11	0.65	0.02	0.93
sTNFR1	-0.53	0.02	-0.22	0.39	-0.16	0.52	-0.04	0.87	0.20	0.44	0.79	0.0002	–	–
Non-ICU	r	p	r	p	r	p	r	p	r	p	r	p	r	p
TNF-α	-0.20	0.28	0.13	0.48	-0.21	0.27	0.01	0.95	-0.10	0.58	0.007	0.97	0.24	0.20
sTNFR1	0.19	0.31	-0.28	0.14	-0.22	0.24	-0.07	0.69	0.16	0.42	0.42	0.02	–	–
Healthy subjects	r	p	r	p	r	p	r	p	r	p	r	p	r	P
sTNFR1	–	–	–	–	–	–	–	–	–	–	0.9154	<0.0001	–	–

R, regression.

## Discussion

In this pilot study we investigated the serum levels of sTNFRI and TNF-α in COVID-19 patients who either required ICU treatment or patients with milder disease who were not treated within the ICU. We found elevated levels sTNFR1 in ICU-treated COVID-19 patients compared to non-ICU-treated patients and healthy controls. In contrast, serum TNF-α levels were higher in COVID-19 patients compared to healthy controls with no difference according to COVID-19 severity. Most (16/17) ICU patients died, were older and had elevated serum LDH levels compared to non-ICU patients. There was a positive correlation between serum sTNFR1 levels in COVID-19 patients with age and a negative correlation between sTNFR1 and ESR in COVID-19 patients treated in the ICU.

A cytokine storm with uncontrolled raised levels of inflammatory mediators is reported in COVID-19 infected patients. As such, elevated levels of some inflammatory cytokines are proposed as predictors of survival and of severity in patients treated within ICU (7). However, some severe COVID-19 subjects express low blood cytokine levels due to immune cell exhaustion (20–22). Elucidation of a possible predictor of COVID-19 severity and survival of patients at admission to ICU is important as this will influence treatment options.

sTNFR1 and sTNFR2 are the circulating forms of their membrane bound comparability (mTNFR1 and mTNFR2) which are required for TNF-α signalling *via* different pathways. Transmembrane forms of TNF-α and its receptors are cleaved by the cell membrane-anchored proteinase ADAM17 resulting in remarkable increase serum levels of sTNFR1 and 2. Neirynck et al. reported that sTNFR1 and sTNFR2 are independently associated to all-cause mortality or an elevated risk for cardiovascular events in advanced chronic kidney disease (CKD) irrespective of the cause of kidney disease (23). In addition, Nishiga and colleagues reported an association between COVID-19 and cardiovascular disease (24). Both cardiovascular and kidney diseases are risk factors and increase mortality in COVID-19 patients.

In addition, immunosuppressive cytokines such as sTNF, IL-1β, TGF-β and IL-10 can abrogate immune functions (25, 26). Moreover, sTNF induces the proliferation and differentiation of myeloid precursors into myeloid-derived suppressor cells (MDSCs) following activation of pSTAT3 (27) and the numbers of MDSCs are elevated in the blood of COVID-19 patients (28). This provides a mechanism by which the increased levels of serum sTNF in ICU patients may drive more severe disease.

Elevated levels of sTNFR1 in the plasma of COVID-19 patients with severe disease have recently been reported (29). This study examined similar numbers of patients to that reported here but also demonstrated increased levels of sTNFR1 in non-severe COVID-19 patients compared with healthy control subjects but these levels were significantly lower than those seen in ICU patients. Despite reporting the expression of a number of other inflammatory mediators and on metabolic enzyme levels, this study did not report TNF-α levels. The differences seen in sTNFR1 expression between our study may reflect the treatment regimens for these patients or the precise time of sampling compared to onset of disease. The McElvaney study also reported that sTNFR1 levels were lower in a control group of non-COVID-19 ICU patients. This was not studied in our cohort of subjects. Together, the data suggest that the levels of serum sTNFR1 upon admission may be indicated of disease severity and, together with elevated TNF-α levels, may impact upon patient’s survival. A larger multi-centre analysis is needed to confirm this. Comparison of the ICU and non-ICU patient groups in healthy control groups indicated that there was significant difference between the older healthy group and the non-ICU patients.

In this study, we showed a negative relationship of sTNFR1 and ESR. Sharma et al. reported a positive relationship between ESR and TNF-α, sTNFR1 and sTNFR2 in chronic heart failure before receiving ACE inhibitors (30). ICU-admitted patients receive antivirals and corticosteroids. The negative correlation observed in this study may reflect the impact of these treatments on these measures or an abnormality in TNF-α regulatory mechanisms induced by COVID-19 infection.

In summary, our data indicates that high serum levels of sTNFR1 are associated with mortality of severe COVID-19 patients within ICU. Future studies should examine whether lower levels of systemic sTNFR1 at admission may indicate a better disease outcome.

## Data Availability Statement

The raw data supporting the conclusions of this article will be made available by the authors, without undue reservation.

## Ethics Statement

The studies involving human participants were reviewed and approved by Masih Daneshvari Hospital ethical Board. The patients/participants provided their written informed consent to participate in this study.

## Author Contributions

EM, NR and ND did experiments. EM wrote the draft of paper. PT, HJ, SH, MM, MR and AM provided the patients and samples. DM and IA revised the paper. IA approved the final version as corresponding author. All authors contributed to the article and approved the submitted version.

## Funding

This study was supported by the authors own funds. The ELISA kits were a gift from GF. IA is supported by the EPSRC (EP/T003189/1), the UK MRC (MR/T010371/1) and by the Wellcome Trust (208340/Z/17/Z) and the Jameel Community Fund.

## Conflict of Interest

The authors declare that the research was conducted in the absence of any commercial or financial relationships that could be construed as a potential conflict of interest.
